# Advances in Feline Panleukopenia Virus Vaccines: Immunological Mechanisms, Current Challenges, and Future Perspectives

**DOI:** 10.3390/v18070750

**Published:** 2026-07-07

**Authors:** Shiqiang Zhu, Weiwei Wang, Huakai Wang, Yuqiang Zhang, Liang Zhao, Wei Xiong

**Affiliations:** 1 Food Laboratory of Zhongyuan, College of Food Science and Technology, Henan University of Technology, Zhengzhou 450001, China; sqzhu92@163.com (S.Z.); bingzhi213608@163.com (W.W.); huakaiwhk@163.com (H.W.); zhangyuqiangyu@163.com (Y.Z.); lzhao@cau.edu.cn (L.Z.); 2 Department of Nutrition and Health, China Agricultural University, Beijing 100083, China

**Keywords:** feline panleukopenia virus, FPV vaccines, vaccine immunology, maternal antibody interference, probiotic-based vaccine vectors, mucosal immunity, viral vaccine development

## Abstract

Feline panleukopenia is a highly contagious and often fatal disease in cats caused by the feline panleukopenia virus (FPV), a member of the Parvoviridae family. Despite the widespread use of vaccination, FPV remains a significant threat to both domestic and wild felid populations worldwide, particularly in young or unvaccinated animals. Effective vaccination strategies are therefore essential for controlling the disease and reducing mortality. Current vaccines, including modified live and inactivated vaccines, have demonstrated substantial efficacy in inducing protective immunity; however, several challenges remain, such as maternal antibody interference, vaccine failure, and safety concerns in certain animal populations. Recent advances in vaccine technology have spurred the development of next-generation FPV vaccines, including recombinant vectors, DNA vaccines, virus-like particle (VLP) vaccines, and novel delivery platforms. Among these, probiotic-based vaccine vectors have garnered growing interest due to their favorable safety profiles, mucosal immunogenicity, and suitability for oral administration. These systems may provide innovative approaches for inducing both systemic and mucosal immune responses against FPV. This review summarizes the current understanding of the immunological mechanisms underlying protection against FPV infection and discusses the progress made in FPV vaccine development. Furthermore, it highlights the major challenges associated with current vaccination strategies and explores emerging vaccine platforms, including probiotic vector-based vaccines, as promising tools for future disease control. Improved vaccine design and optimized immunization strategies will be crucial for enhancing the prevention of feline panleukopenia in the future.

## 1. Introduction

Feline panleukopenia is a highly contagious and potentially fatal viral disease affecting domestic and wild felids worldwide [1,2,3]. The disease is caused by the feline panleukopenia virus (FPV), a small non-enveloped single-stranded DNA virus belonging to the family *Parvoviridae* [4,5,6]. FPV primarily targets rapidly dividing cells, particularly those in the intestinal crypts, bone marrow, and lymphoid tissues, leading to severe clinical manifestations such as leukopenia, enteritis, dehydration, and high mortality rates, especially in kittens [5,7]. Despite the widespread availability of vaccines, FPV remains a persistent threat to feline populations due to its remarkable environmental stability, remaining infectious in the environment for days to weeks, and its efficient transmission through contaminated environments and fomites [8,9,10,11]. Epidemiological surveillance has revealed the continuous circulation of FPV worldwide, with distinct genetic lineages and novel VP2 variants—particularly mutations at residues 426, 564, and 568—emerging in regions such as China and Europe [12,13,14]. Some of these mutations occur within critical neutralizing epitopes, raising concerns about potential antigenic drift that may impact the cross-protective efficacy of current vaccines [15,16]. Vaccination has therefore become the cornerstone strategy for preventing feline panleukopenia and controlling outbreaks in both companion animal populations and animal shelters. Vaccination has therefore become the cornerstone strategy for preventing feline panleukopenia and controlling outbreaks in both companion animal populations and animal shelters. Nevertheless, several challenges continue to limit the effectiveness of current vaccination strategies, including maternal antibody interference, vaccine failure in certain conditions, and the need for improved safety and immunogenicity profiles [9,17,18]. In recent years, progress in immunology and vaccine technology has spurred the exploration of novel platforms, including recombinant vaccines, VLPs, and innovative delivery systems such as probiotic-based vectors [6,19,20,21,22,23]. A comprehensive understanding of the immunological mechanisms that confer protective immunity against FPV, alongside the development of improved vaccination strategies, is therefore essential for advancing disease control [6,24].

Vaccination has long been recognized as the most effective measure for preventing FPV infection and reducing disease-associated mortality [9]. Currently available vaccines mainly include modified live vaccines (MLVs) and inactivated vaccines, both of which have demonstrated considerable efficacy in inducing protective immunity in cats [25,26]. MLVs are widely used due to their ability to stimulate strong and long-lasting immune responses, whereas inactivated vaccines are often considered safer for specific populations, such as pregnant or immunocompromised animals [6,26]. Despite the success of these conventional vaccines, several limitations remain. One of the major obstacles in feline vaccination programs is the interference of maternally derived antibodies, which can significantly reduce vaccine efficacy in young kittens [27]. Additionally, concerns regarding vaccine safety, incomplete immune protection, and the persistence of FPV in contaminated environments continue to pose challenges for disease control. In response to these issues, increasing research efforts have focused on the development of next-generation FPV vaccines, including recombinant vector vaccines, DNA vaccines, virus-like particle (VLP) vaccines, and other innovative vaccine platforms aimed at improving immunogenicity, safety, and delivery efficiency.

Among the emerging strategies for viral vaccine development, probiotic-based vaccine delivery systems have recently attracted growing interest [28,29]. Certain probiotic microorganisms, particularly species of Lactobacillus and Bifidobacterium, have been explored as potential vaccine vectors due to their favorable safety profiles, ability to colonize mucosal surfaces, and capacity to stimulate both mucosal and systemic immune responses [19]. These properties make probiotic vectors promising tools for the development of novel oral or mucosal vaccines against viral pathogens. Although the application of probiotic-based vaccine platforms has been investigated in several viral diseases, their potential use in FPV vaccination remains an emerging and relatively underexplored field. In this review, we summarize the current understanding of the immunological mechanisms underlying protective immunity against FPV and discuss recent advances in FPV vaccine development. Particular attention is given to novel vaccine technologies and emerging delivery platforms, including probiotic-based vectors, as well as the key challenges and future perspectives for improving vaccination strategies against feline panleukopenia.

## 2. Virological and Pathogenic Characteristics of FPV

### 2.1. Genome Organization and Viral Structure

Feline panleukopenia virus (FPV) is a non-enveloped, single-stranded DNA (ssDNA) virus belonging to the *Parvoviridae* family, genus *Protoparvovirus* [12,30]. The viral genome is approximately 5.1 kb in length and encodes two major functional regions: the non-structural (NS) and structural (VP) proteins [31]. The NS proteins, primarily NS1 and NS2, are essential for viral replication, genome packaging, and cytotoxicity [32,33]. By contrast, the structural proteins VP1 and VP2 assemble to form the icosahedral capsid [34,35]. VP2 constitutes the major capsid component (approximately 90%) and is responsible for host cell receptor recognition and antigenicity, while VP1 comprises the remaining 10% and contributes unique N-terminal sequences critical for cell infectivity, including phospholipase A2 (PLA2) enzymatic activity [32,36,37]. VP2 constitutes the major capsid component and is responsible for host cell receptor recognition and antigenicity, while VP1 contributes unique N-terminal sequences required for viral infectivity and nuclear entry [38].

The VP2 protein contains the primary antigenic determinants recognized by neutralizing antibodies, making it the principal target for vaccine design [6,39,40]. Conformational epitopes on VP2 mediate protective humoral immunity, whereas conserved regions among FPV strains enable cross-protection [40,41,42,43]. The capsid structure exhibits high stability, which contributes to the virus’s resistance to environmental conditions and complicates disinfection measures [43,44,45]. Understanding the molecular features and antigenic topology of FPV is therefore critical for rational vaccine development, particularly for next-generation subunit or recombinant vaccine platforms.

### 2.2. Viral Replication and Host Tropism

**Figure 1:**
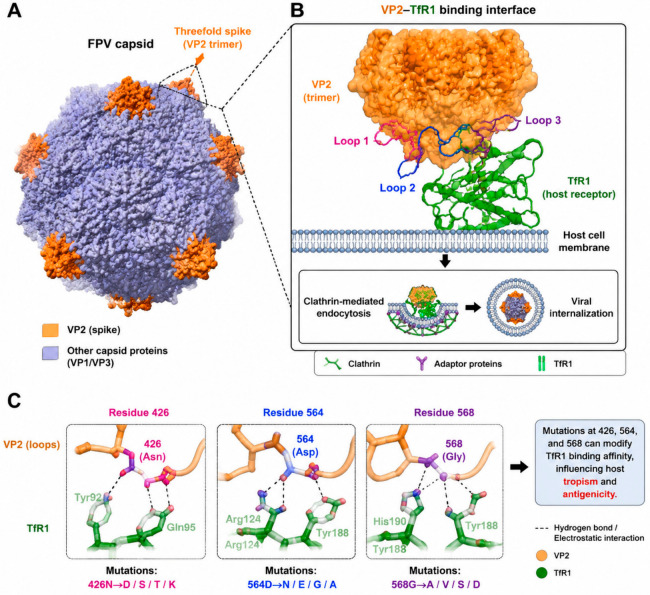
Schematic representation of FPV VP2-TfR1 receptor binding interface. (**A**) Structure of the FPV capsid showing the threefold spike protrusions formed by VP2 trimers. (**B**) Enlarged view of the VP2 surface-exposed loops (loops 1, 2, and 3) interacting with the host transferrin receptor type 1 (TfR1) on the cell membrane. (**C**) Key amino acid residues (e.g., 426, 564, 568) within the binding interface that are subject to mutation and influence host tropism and antigenicity [18].

Following cell attachment, FPV replication is strictly dependent on host cellular machinery activated during the S-phase of the cell cycle, thereby restricting viral propagation to mitotically active cells [1,11]. Infection is initiated through the binding of the viral capsid protein VP2 to transferrin receptor type 1 (TfR1) on actively dividing cells, including intestinal crypt epithelial cells, bone marrow progenitors, and lymphoid tissues [11,45,46,47,48,49,50]. Structural analyses have mapped the receptor-binding interface to the threefold spike of the capsid, involving surface-exposed loops of VP2 (Figure 1). Mutations within this region can modify transferrin receptor (TfR) binding affinity, consequently influencing host tropism and antigenicity [35,49,51].

FPV exhibits a highly restricted host range, primarily infecting domestic cats (Felis catus) and certain wild felids, with occasional cross-species infections in related carnivores [52]. Following receptor engagement, the virus–TfR complex is internalized via clathrin-mediated endocytosis and transported to perinuclear endosomal compartments [49]. After endosomal escape, the capsid traffics to the nucleus, where uncoating occurs and the single-stranded DNA genome is converted into a double-stranded replicative form [45,48,51,53]. Viral replication is driven by host DNA polymerases, while the nonstructural protein NS1 orchestrates genome amplification, capsid assembly, and the induction of cytopathic effects [54,55]. NS1 functions as a multifunctional helicase that binds the origin of replication, unwinds DNA, and recruits host polymerases, while also contributing to cell cycle arrest and cytotoxicity, ultimately leading to host cell death. Amino acid substitutions in NS1, particularly within conserved functional domains (e.g., residues 60, 443, 544–545), may influence replication efficiency and are associated with strain-specific differences in virulence [35,51]. Following genome packaging, progeny virions are released upon cell lysis, perpetuating the infectious cycle.

FPV preferentially targets rapidly proliferating cells, including those in the intestinal crypts, bone marrow, and lymphoid tissues, resulting in severe leukopenia, enteritis, and immunosuppression [56]. This cellular tropism underlies the high morbidity observed in young kittens, as their rapidly dividing tissues and immature immune systems render them particularly susceptible [48]. Maternal antibodies provide transient protection but can interfere with vaccine efficacy, representing a critical consideration in immunization scheduling [9,27,57].

### 2.3. Pathogenesis of FPV Infection

The pathogenesis of feline panleukopenia virus (FPV) infection is a complex, multifactorial process, largely dictated by the virus’s strict requirement for mitotically active cells, its distinct cellular tropism, and the host’s age and immune status [11]. These factors collectively determine a spectrum of clinical outcomes, ranging from subclinical infection to severe, often fatal disease, typically manifesting as the classical triad of panleukopenia, enteritis, and, in certain cases, cerebellar hypoplasia [11,43].

FPV exhibits rapid replication in actively dividing cells, resulting in pronounced cytopathology across multiple tissues [4,11]. Intestinal crypt epithelial cells represent the primary site of viral replication, where infection induces severe villous atrophy, impaired nutrient absorption, and diarrhea [3,4]. Concurrently, infection of bone marrow progenitor cells leads to leukopenia, immunosuppression, and increased vulnerability to secondary infections. In neonatal kittens, FPV can traverse the placenta or target cerebellar precursor cells, culminating in cerebellar hypoplasia and irreversible neurological deficits [9,11].

## 3. Immune Responses to FPV Infection

### 3.1. Humoral Immune Response Overview of Innate and Adaptive Immune Responses

**Figure 2:**
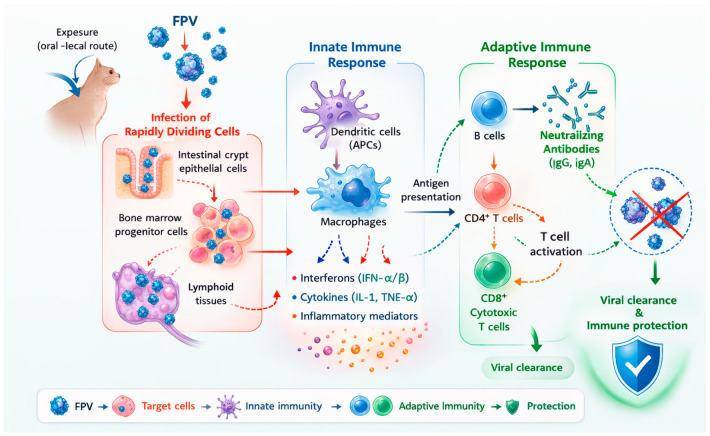
Innate and Adaptive Antiviral Responses in Cats with Strong Immunity Against FPV.

Upon FPV infection, the host mounts a coordinated immune defense aimed at limiting viral spread and clearing infected cells. The innate immune response is rapidly activated upon viral entry, with antigen-presenting cells—including dendritic cells and macrophages—recognizing viral components and producing type I interferons and pro-inflammatory cytokines. These mediators not only suppress early viral replication but also orchestrate the subsequent adaptive immune response. B lymphocyte activation drives the production of virus-specific neutralizing antibodies, which are essential for preventing viral dissemination and reinfection. Simultaneously, T lymphocytes, comprising CD4^+^ helper and CD8^+^ cytotoxic subsets, facilitate viral clearance through cytokine-mediated support and direct cytolysis of infected cells. The interplay between innate and adaptive immunity ultimately shapes the infection outcome, promoting viral elimination and long-term protective immunity (Figure 2) [11,58,59]. The following sections detail the specific roles of humoral, cellular, and mucosal immunity in protection against FPV.

### 3.2. Humoral Immune Response

Humoral immunity is central to controlling FPV infection and conferring long-term protection [9]. Upon viral exposure, B cells differentiate into plasma cells that produce neutralizing antibodies (nAbs), primarily directed against the VP2 capsid protein [21]. These antibodies block viral entry by inhibiting VP2-mediated receptor binding, thereby preventing subsequent replication [60,61,62].

Neutralizing antibody (nAb) titers are strongly correlated with protection against clinical disease. In vaccinated cats, sustained high levels of VP2-specific nAbs confer long-term immunity that can persist for several years, and are therefore regarded as a key correlate of vaccine-induced protective efficacy. Maternal antibodies transferred via colostrum confer passive immunity to kittens but can interfere with active immunization, necessitating carefully timed vaccination schedules to ensure optimal seroconversion [63,64].

From a vaccine design perspective, eliciting robust and durable humoral responses against VP2 epitopes is a primary goal. Live-attenuated vaccines efficiently induce high-titer systemic and mucosal antibodies, whereas subunit and virus-like particle (VLP) vaccines focus on presenting VP2 in its native conformation to stimulate neutralizing responses without the risks associated with viral replication [18]. Strategies enhancing antibody affinity maturation and memory B cell formation are critical for extending long-term protection, particularly in the face of maternal antibody interference and emerging viral variants [65,66].

### 3.3. Cellular Immunity

Cell-mediated immunity is indispensable for controlling FPV infection, particularly for the clearance of infected cells and the resolution of established disease [11,67]. CD8^+^ cytotoxic T lymphocytes (CTLs) recognize viral peptide epitopes presented on MHC class I molecules of infected host cells, leading to the targeted lysis of FPV-infected intestinal crypt epithelial cells and bone marrow progenitors [68]. This cytolytic activity is essential for eliminating virus reservoirs, especially in tissues where neutralizing antibodies may have limited access, such as the intestinal mucosa and lymphoid organs. Beyond direct cytolysis, CD8^+^ T cells also produce antiviral cytokines, including IFN-γ and TNF-α, which contribute to the suppression of viral replication [69].

CD4^+^ helper T cells play a dual role in the anti-FPV immune response. First, they provide critical co-stimulatory signals for B cell activation, immunoglobulin isotype switching, and affinity maturation within germinal centers, thereby ensuring the robust production of high-affinity VP2-specific neutralizing antibodies [21,70]. Second, CD4^+^ T cells orchestrate the cellular arm of immunity by secreting Th1-polarizing cytokines, such as IFN-γ and IL-2, which promote CD8^+^ T cell proliferation, enhance natural killer (NK) cell activity, and activate macrophage-mediated viral clearance [68,71,72]. The balance between Th1 and Th2 responses is particularly relevant for vaccine design, as vaccines that preferentially induce a Th1-skewed response may offer superior protection against intracellular pathogens like FPV.

In the context of vaccine design, inducing balanced CD4^+^ and CD8^+^ T cell responses are crucial for durable protection. While live-attenuated vaccines can naturally elicit strong cellular immunity, subunit, VLP, and viral-vectored vaccines often require adjuvants or delivery systems that enhance T cell priming [22,69,73,74]. Optimizing antigen presentation to both MHC class I and II pathways ensure coordinated humoral and cellular responses, thereby promoting rapid viral clearance upon challenge and reducing clinical severity [75].

Effective vaccines thus aim to elicit multifaceted immunity, combining neutralizing antibodies with potent CD8^+^ CTL responses, supported by CD4^+^ T cell help, to confer both immediate and long-term protection against FPV infection [6,23,76].

### 3.4. Mucosal Immunity

FPV is primarily transmitted via the fecal–oral route, rendering mucosal immunity a critical component of the protective host response [17,29]. Secretory IgA (sIgA) antibodies at the intestinal mucosa can neutralize viral particles prior to epithelial cell infection, thereby limiting viral replication and dissemination at the portal of entry [77,78]. Effective induction of mucosal sIgA not only provides local protection but also contributes to systemic immunity by facilitating antigen sampling by dendritic cells and subsequent priming of lymphoid tissues [79,80]. Importantly, conventional parenteral vaccines predominantly elicit systemic IgG responses and often fail to induce robust mucosal immunity, leaving the gastrointestinal tract vulnerable to initial viral challenge. This limitation has motivated the exploration of oral and mucosal vaccine strategies, including probiotic-based vaccine vectors, which can deliver antigens directly to the intestinal mucosa, stimulate local IgA production, and concurrently prime systemic humoral and cellular responses. Consequently, enhancing mucosal immunity represents a pivotal strategy in the design of next-generation FPV vaccines, providing both direct protection at the portal of viral entry and synergistic systemic immune responses [17].

### 3.5. Maternal Antibody Interference

**Figure 3:**
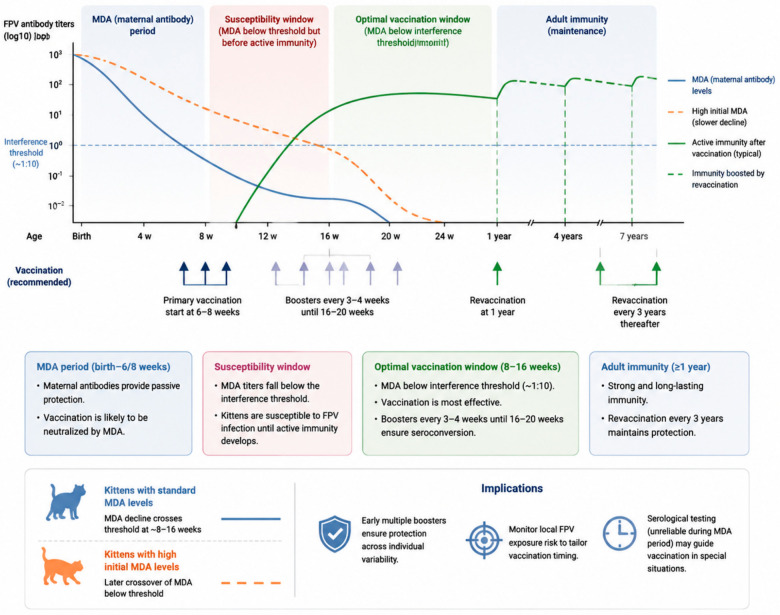
Schematic timeline of FPV vaccination strategies illustrating maternal antibody interference and optimal vaccination windows [18].

Maternal antibodies, acquired transplacentally or via colostrum, play a crucial role in early neonatal protection against FPV [63]. High titers of maternally derived antibodies (MDA) in young kittens are known to interfere with vaccine-induced immunity by neutralizing vaccine antigens and thereby preventing the activation of the neonatal B-cell repertoire; consistent with this mechanism, Jakel et al. (2012) explicitly demonstrated that MDA suppress the establishment of active immunity, with failure of seroconversion occurring even at titers as low as ≥1:10 (Figure 3) [18]. This interference can significantly delay the onset of effective protective immunity, creating a critical window of susceptibility to FPV infection [27]. The magnitude and duration of maternal antibody-mediated interference vary depending on the maternal vaccination status, litter size, and the timing of colostrum intake [81]. To overcome this limitation, vaccination schedules must be carefully optimized, typically through multiple doses at strategic intervals to coincide with the waning of maternal antibodies [18]. Novel vaccination strategies, including recombinant, DNA, and mucosal/probiotic-based vaccines, are being explored to circumvent maternal antibody interference by enhancing immunogenicity or targeting mucosal immune induction. A comprehensive understanding of maternal antibody kinetics and their impact on vaccine responsiveness is therefore essential for the rational design of effective FPV immunization programs in neonatal and young cats.

This schematic illustrates the dynamic interplay between declining maternal antibodies (MDA, blue curve) and vaccine-induced immunity (red curve) throughout early life (Figure 3). The yellow-shaded susceptibility window represents the critical gap when MDA drops below protective levels before active immunity develops. Vaccination starts at 6–8 weeks, with boosters every 3–4 weeks until 16–20 weeks to ensure seroconversion. Individual variation in MDA decay (e.g., high initial titers) may shift the optimal vaccination window, supporting an early multi-booster approach. Adult immunity is maintained by revaccination at 1 year and triennially thereafter [18].

## 4. Current FPV Vaccines and Immunization Strategies

### 4.1. Modified Live Vaccines (MLV)

**Figure 4:**
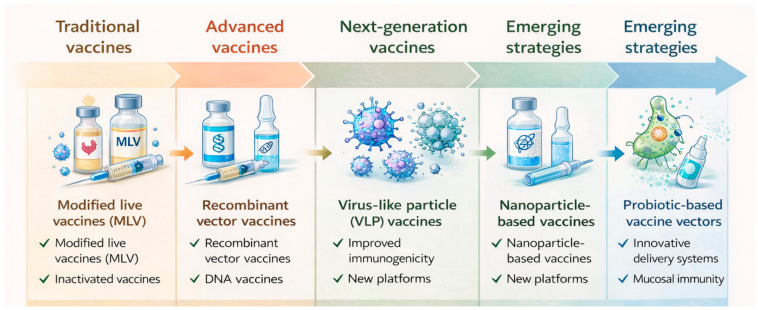
Evolution of vaccine platforms from traditional to emerging strategies, highlighting key types and immunological features.

Modified live vaccines (MLVs) have been widely used for the prevention of feline panleukopenia virus (FPV) due to their robust immunogenicity and ability to induce long-lasting protective responses (Figure 4) [27]. These vaccines contain attenuated FPV strains capable of limited replication in the host, effectively mimicking natural infection and stimulating both humoral and cellular immunity [25]. Studies have demonstrated that MLVs elicit high titers of neutralizing antibodies, induce CD4^+^ and CD8^+^ T cell responses, and provide durable protection that can persist for months to years in domestic cats [82,83].

Despite these advantages, safety concerns remain a critical consideration in the use of MLVs. Replication-competent vaccine strains can pose risks in immunocompromised or pregnant cats, potentially leading to transient clinical signs or, in rare cases, vaccine-induced disease [84,85,86]. For instance, vaccine-associated adverse events, including mild fever, lethargy, and gastrointestinal signs, have been reported in immunosuppressed felines, and severe outcomes such as vaccine-induced panleukopenia have been documented in neonatal kittens with immature immune systems. Similarly, recombinant viral vectors, although generally safer due to replication-defective designs, may still trigger unintended inflammatory responses or insertional mutagenesis in susceptible hosts. Recent safety studies emphasize the need for rigorous preclinical evaluation of these platforms before their widespread use in high-risk populations. Additionally, interference from maternal antibodies in kittens can reduce vaccine efficacy, necessitating carefully timed booster schedules [27]. Recent research has therefore focused on optimizing MLV formulations, evaluating immunization intervals, and exploring complementary strategies, such as mucosal or recombinant vaccines, to enhance safety and overcome the limitations associated with maternal antibody interference. Overall, while MLVs remain the cornerstone of FPV prevention, their use must be balanced with considerations of host susceptibility and programmatic vaccination strategies.

Recent reports have documented adverse events following MLV administration, including transient leukopenia, fever, and gastrointestinal signs, particularly in kittens with subclinical infections or underlying immunosuppression. In rare cases, vaccine-associated parvovirus disease has been observed in severely immunocompromised cats, underscoring the need for careful risk-benefit assessment. For recombinant viral vectors, although replication-defective designs minimize reversion risk, vector-induced inflammatory responses and potential off-target antigen expression remain safety considerations that require further investigation in feline models.

### 4.2. Inactivated Vaccines

Inactivated FPV vaccines (Figure 4), also known as killed vaccines, are produced by chemically or physically inactivating the virus while preserving its antigenic structure. These vaccines are unable to replicate in the host, which eliminates the risk of vaccine-induced infection, making them particularly suitable for immunocompromised or pregnant cats [64]. Administration of inactivated FPV vaccines primarily induces humoral immunity, leading to the production of neutralizing antibodies against VP2 and other protective epitopes [87]. However, compared to modified live vaccines, inactivated vaccines generally elicit weaker immune responses and provide a shorter duration of immunity [25,88]. Consequently, multiple doses or booster vaccinations are required to achieve and maintain protective antibody titers, particularly in kittens or high-risk populations. Despite these limitations, inactivated FPV vaccines remain an important tool for FPV prevention when safety considerations outweigh the need for rapid or strong immune induction, such as in pregnant queens or immunologically vulnerable animals [89].

### 4.3. Current Vaccination Guidelines in Cats

Effective vaccination programs against feline panleukopenia virus (FPV) require carefully designed schedules that account for both the age of the kitten and the potential interference of maternally derived antibodies. Standard protocols typically initiate primary vaccination between 6 and 8 weeks of age, followed by additional doses every 3–4 weeks until 16–20 weeks of age to ensure the development of protective immunity. Booster vaccinations are generally recommended 1 year after the primary series and subsequently every 3 years to maintain long-term antibody titers in adult cats (Figure 3) [9,90].

In high-risk populations, such as shelters or catteries with frequent introductions of naive kittens, vaccination schedules may be accelerated or intensified to rapidly establish herd immunity. Special consideration is given to kittens with high maternal antibody levels, as these antibodies can neutralize vaccine antigens and reduce the efficacy of early immunization; in such cases, the timing of initial and booster doses may be adjusted accordingly. Overall, adherence to recommended vaccination schedules and booster programs is essential to achieve sustained protection against FPV, minimize susceptibility windows, and prevent outbreaks, particularly in high-density or immunologically vulnerable populations [63,91,92].

## 5. Advances in Next-Generation FPV Vaccines

Next-generation FPV vaccines are being developed to overcome key limitations of conventional modified-live and inactivated vaccines, including maternal antibody interference, safety risks in neonates and immunocompromised animals, and weak cellular immunity. Recent progress in molecular biology and nanotechnology has facilitated the creation of novel platforms with improved immunogenicity, safety profiles, and delivery precision. Among these, recombinant viral vectors, DNA vaccines, virus-like particles (VLPs), and nanoparticle-based vaccines have emerged as promising candidates for both prophylactic and therapeutic applications (Figure 4) [6,17,24,91]. Similarly, a raccoon poxvirus (RCN) vector expressing VP2 has been shown to induce protective neutralizing antibodies in cats, representing an alternative poxvirus-based platform with a distinct safety profile [93,94]. More recently, a recombinant feline herpesvirus type 1 (FHV-1) vector has been developed as a multivalent vaccine candidate. This vector not only expresses the FPV VP2 protein but also retains the immunogenicity of FHV-1 antigens, thereby offering dual protection against both FPV and FHV-1 infection in a single immunization [6,28].

Recombinant viral vector vaccines employ replication-deficient, non-pathogenic viruses—such as adenoviruses, poxviruses, or herpesviruses—to deliver FPV antigens, primarily the major capsid protein VP2, and induce robust immune responses. These vectors efficiently elicit both CD4^+^ and CD8^+^ T-cell responses in addition to potent neutralizing antibodies. Certain vector systems can overcome maternal antibody interference, enabling effective vaccination in young kittens, while replication-defective design minimizes safety concerns. Preclinical studies have demonstrated that adenovirus-vectored VP2 vaccines confer complete protection against virulent FPV challenge, with viral clearance mediated by coordinated humoral and cellular immunity [6,19,21,24,58]. Despite these advantages, recombinant viral vector vaccines face challenges including pre-existing vector immunity in the target population, which can reduce transgene expression and immunogenicity. Manufacturing complexity and higher production costs compared to conventional vaccines also limit their widespread adoption. Furthermore, the potential for vector genome integration and long-term safety in cats remain to be fully evaluated.

DNA vaccines represent a promising strategy for controlling feline panleukopenia virus (FPV) infection by encoding viral antigens within plasmid constructs that are expressed in host cells, thereby eliciting broad immune responses [95,96]. These vaccines offer intrinsic thermostability and can be rapidly adapted to emerging FPV variants [97,98]. Beyond humoral immunity, DNA vaccination can induce robust cytotoxic CD8^+^ T cell responses, a critical component for viral clearance [99,100,101]. Strategies such as co-administration with molecular adjuvants or the application of electroporation have been shown to further enhance immunogenicity in analogous systems [102,103]. Although FPV VP2 DNA vaccines have yet to be formally reported, insights from parvovirus DNA vaccine platforms suggest that, particularly when combined with immunostimulatory molecules such as cytokine genes, these vaccines hold considerable potential to confer protection in feline models against FPV-induced leukopenia and intestinal lesions [23,58,93,104]. However, DNA vaccines have historically shown poor immunogenicity in large animals, including cats, due to inefficient cellular uptake and nuclear translocation. The requirement for specialized delivery devices, such as electroporation, complicates field application. Additionally, concerns regarding plasmid integration and the induction of anti-DNA antibodies warrant further safety evaluation.

Virus-like particle (VLP) vaccines are self-assembling protein structures that structurally mimic FPV virions without containing viral genomes, thus providing an excellent safety profile [22]. VLPs display VP2 conformational epitopes in their native configuration, eliciting strong neutralizing antibody responses and activating CD4^+^ and CD8^+^ T-cell immunity [23]. Their versatility allows incorporation of multiple antigens or epitopes from diverse FPV strains, broadening protective coverage [22,105]. Studies in kittens indicate that VP2 VLPs generate durable immunity, making them highly promising for both prophylactic vaccination and potential therapeutic strategies [20,22,23]. Nonetheless, VLP production often relies on complex eukaryotic expression systems, leading to high manufacturing costs and challenges in large-scale purification. Stability during storage and transportation, as well as the need for potent adjuvants to enhance immunogenicity in neonatal or immunocompromised hosts, remain unresolved issues.

Nanoparticle-based vaccines leverage nanoscale carriers—including liposomes, polymeric nanoparticles, and protein-based nanoparticles—to improve antigen stability, targeted delivery, and immune activation [106]. These systems enhance antigen uptake by dendritic cells, promote cross-presentation to T cells, and can induce robust mucosal IgA responses in addition to systemic IgG, which is particularly important for intestinal FPV infection [107,108]. Nanoparticles can also co-deliver immunostimulatory molecules to modulate Th1/Th2 responses [109]. Recent studies employing biodegradable and surface-engineered nanocarriers have demonstrated enhanced viral clearance and sustained immunity in feline models, highlighting the potential of nanotechnology to advance next-generation FPV vaccines [110,111].

Despite their promise, nanoparticle-based vaccines face hurdles in clinical translation, including batch-to-batch consistency, long-term stability, and potential toxicity of certain nanocarrier materials. Regulatory pathways for nanoparticle-based biologics are still evolving, and comprehensive safety and toxicology studies in target species are lacking.

In summary, these next-generation platforms differ markedly in their developmental maturity, production complexity, and practical applicability (Table 1). While recombinant viral vectors and VLPs offer potent immunogenicity, they generally require parenteral administration and are costly to produce. DNA vaccines provide thermostability and rapid adaptability but face delivery hurdles in large animals. In contrast, probiotic-based vectors enable non-invasive oral delivery and superior mucosal induction, yet their scalability, antigen stability, and regulatory pathways as live biotherapeutic products (LBPs) remain to be fully established. This comparative perspective underscores that no single platform is likely to address all FPV control challenges; rather, the optimal approach may depend on the specific target population and epidemiological context.

## 6. Probiotic-Based Vaccine Vectors for FPV

### 6.1. Probiotic Bacteria as Vaccine Delivery Systems

**Figure 5:**
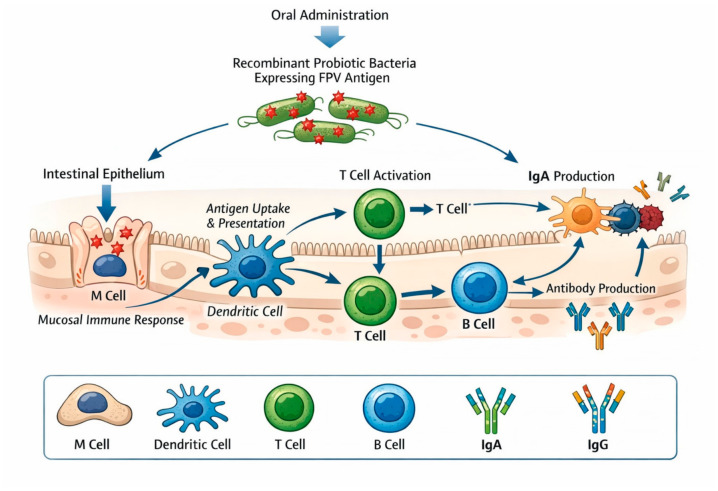
Mechanism of mucosal immune activation by oral administration of recombinant probiotic bacteria expressing FPV antigen.

Probiotic bacteria, particularly strains of Lactobacillus and Bifidobacterium, have emerged as promising vaccine delivery vectors due to their inherent safety, ability to survive gastrointestinal transit, and potential to stimulate mucosal immune responses [112,113,114]. As depicted in Figure 5, these microorganisms can be genetically engineered to express viral antigens, such as the VP2 protein of FPV, on their surface or secrete them into the intestinal lumen [19]. Oral administration of recombinant probiotics allows direct antigen presentation to the gut-associated lymphoid tissue (GALT), providing both local and systemic immune activation [115,116]. Compared to traditional parenteral vaccines, probiotic vectors offer a non-invasive route of administration and may be particularly suitable for neonatal kittens or immunocompromised cats, in which conventional vaccines carry higher safety risks [19,89,117,118].

### 6.2. Mechanisms of Immune Activation by Probiotic Vectors

Upon colonization of the intestinal mucosa, recombinant probiotic bacteria interact with specialized M cells and dendritic cells, facilitating antigen uptake and processing (Figure 5) [119,120]. This interaction leads to activation of both mucosal and systemic immunity [121]. At the mucosal level, secretory IgA antibodies are produced, providing the first line of defense against FPV entry [119,122]. Concurrently, antigen presentation triggers B cell-mediated production of systemic IgG antibodies and the activation of CD4^+^ helper and CD8^+^ cytotoxic T cells, contributing to viral clearance [119,123,124]. The coordinated stimulation of humoral and cellular immunity by probiotic vectors provides a comprehensive protective response, which may complement or enhance the efficacy of conventional vaccines [118]. Mechanistic studies in other viral models have demonstrated that probiotic-mediated antigen delivery can induce durable immunity with lower risk of adverse effects, supporting the rationale for FPV applications [120,125].

### 6.3. Application and Potential of Probiotic-Based FPV Vaccines

Probiotic-based vaccine platforms have been evaluated against several viral pathogens, including influenza virus, rotavirus, and coronavirus, consistently demonstrating their capacity to induce both mucosal and systemic immune responses [126,127,128]. These studies provide compelling proof-of-concept for their application to FPV vaccination [19]. Key advantages over conventional live-attenuated vaccines include non-invasive oral administration, superior induction of mucosal IgA, and an inherently safer profile, particularly for use in neonatal or immunocompromised animals [19,129,130]. Moreover, probiotic vectors can be engineered to express multiple viral antigens or combined with adjuvants to further enhance immunogenicity [128,130,131,132]. A recent proof-of-concept study has directly applied this platform to FPV by constructing a recombinant Lactobacillus plantarum strain that displays the FPV VP2 protein on its surface [19]. Oral administration of this recombinant probiotic to mice and cats induced significant VP2-specific mucosal secretory IgA (sIgA) responses in the intestine and systemic IgG antibodies in serum. Moreover, vaccinated animals exhibited enhanced T-cell proliferation and cytokine production, indicating the activation of cellular immunity. Crucially, cats orally immunized with the recombinant L. plantarum showed reduced viral shedding and milder clinical signs upon FPV challenge, providing the first direct evidence for the protective efficacy of a probiotic-based FPV vaccine [19]. These platforms offer a promising alternative or complement to conventional MLV and inactivated vaccines, particularly for populations where safety and mucosal immunity are priorities [128]. Future investigations should focus on optimizing antigen expression, dosing strategies, and delivery methods to maximize protective efficacy in domestic cats.

To date, direct evidence for probiotic-based FPV vaccines in cats remains limited, with most data derived from murine models or other viral pathogens. Comparative studies in feline models or closely related carnivore species (e.g., ferrets) are urgently needed to validate immunogenicity and protective efficacy. Beyond biological performance, scalability of recombinant probiotic production—including fermentation yield, antigen expression stability, and formulation for oral delivery—poses significant industrial challenges. Regulatory pathways for live biotherapeutic products (LBPs) as vaccines are not yet well-defined in veterinary medicine; obtaining a Biologics License Application (BLA) or equivalent approval will require extensive characterization of strain identity, purity, potency, and safety, as well as demonstration of batch-to-batch consistency and long-term stability.

Mechanism of mucosal and systemic immune activation by oral recombinant probiotic vaccines (Figure 5). Following oral administration, recombinant probiotics expressing FPV VP2 antigen are sampled by M cells and transferred to underlying dendritic cells (DCs) and macrophages in gut-associated lymphoid tissue (GALT). Antigen presentation by DCs primes CD4^+^ helper T cells and CD8^+^ cytotoxic T cells, triggering two arms of immunity: (i) Mucosal immunity—B cell differentiation into IgA-producing plasma cells, yielding secretory IgA (sIgA) for local neutralization of FPV; and (ii) Systemic immunity—production of circulating IgG antibodies and activation of CTLs for viral clearance. Thus, probiotic vectors bridge local mucosal defense and systemic protective responses.

## 7. Challenges and Future Perspectives in FPV Vaccination

Despite substantial progress in FPV vaccine development, several challenges persist that limit optimal disease control and delineate priorities for future research. Maternal antibody interference remains a primary concern, as maternal-derived antibodies (MDA) can neutralize vaccine antigens in young kittens, delaying the onset of active immunity and creating a critical window of susceptibility [133]. Vaccine failure can also arise from incomplete immunization coverage, improper administration, or antigenic mismatches between vaccine strains and circulating field variants [134,135,136]. The remarkable environmental stability of FPV further complicates prevention, as the virus can persist on fomites, surfaces, and in shelters for extended periods, increasing the likelihood of exposure even among vaccinated populations. Additionally, viral evolution, including antigenic drift in VP2 and other immunodominant epitopes, may reduce vaccine efficacy over time, necessitating updates to vaccine formulations [13,15,135,136]. Balancing safety and immunogenicity remain critical, particularly in vulnerable populations such as immunocompromised cats, pregnant queens, and neonatal kittens.

To address these multifaceted challenges, next-generation vaccine platforms offer promising solutions. Recombinant viral vectors, DNA vaccines, virus-like particle (VLP) vaccines, nanoparticle-based vaccines, and probiotic-based vectors provide opportunities to enhance both systemic and mucosal immunity while maintaining safety in sensitive populations [21,22,23]. Optimizing vaccination schedules to account for maternal antibody interference, combined with careful herd immunity planning and continuous surveillance of circulating FPV strains, is essential to maximize protection at both individual and population levels [14,16,135]. Furthermore, innovations such as oral or mucosal delivery systems, multi-epitope vaccine design, and incorporation of adjuvants hold potential to improve immunogenicity and broaden protective coverage. Collectively, overcoming current limitations while leveraging these emerging technologies will be crucial for the development of effective, safe, and durable FPV vaccination strategies in domestic cats and high-risk populations.

Future research priorities should include: (i) optimizing antigen display strategies in probiotic vectors, such as surface anchoring versus secretion, to maximize mucosal immune activation; (ii) designing multi-epitope or chimeric antigens that incorporate conserved neutralizing epitopes from diverse FPV strains to broaden protective coverage; (iii) developing immune monitoring strategies in neonates, including non-invasive salivary or fecal IgA detection, to guide individualized vaccination timing in the presence of maternal antibodies; (iv) evaluating novel adjuvants and delivery vehicles, such as lipid nanoparticles or plant-based expression systems, for enhanced stability and immunogenicity; and (v) conducting field studies to assess real-world vaccine performance across different feline populations and environmental settings.

## 8. Conclusions

Vaccination remains the cornerstone of feline panleukopenia virus (FPV) prevention, providing essential protection against a highly contagious and often fatal disease. Traditional vaccines, including modified live and inactivated formulations, have demonstrated substantial efficacy in inducing protective humoral and cellular immune responses, yet limitations such as maternal antibody interference, safety concerns, and incomplete coverage underscore the need for continued improvement. Advances in next-generation vaccine platforms, including recombinant viral vectors, DNA vaccines, virus-like particles, nanoparticle-based vaccines, and probiotic-based delivery systems, offer promising strategies to enhance immunogenicity, induce robust mucosal and systemic immunity, and address safety challenges, particularly in vulnerable populations such as neonatal or immunocompromised cats.

Integrating mechanistic insights into FPV immunology with innovative vaccine design is critical for overcoming existing barriers and optimizing immunization programs. Future research should prioritize the improvement of antigen delivery methods, the design of multi-epitope or broadly protective antigens, and the implementation of immunization schedules that account for maternal antibody kinetics and the specific needs of high-risk populations. By combining traditional approaches with emerging technologies, it is possible to develop safer, more effective, and long-lasting FPV vaccines that ensure both individual protection and herd immunity, ultimately contributing to better feline health worldwide.

## Figures and Tables

**Table 1:** Next-generation FPV vaccine platforms: characteristics, immune mechanisms, and experimental outcomes.

Vaccine Type	Key Features/ Advantages	Immune Mechanisms	Preclinical/Experimental Outcomes	References
Recombinant Viral Vector Vaccines	Non-pathogenic viral carriers (adenovirus, poxvirus, herpesvirus) Can overcome maternal antibody interference High safety (replication-defective)	Elicits strong CD4^+^ and CD8^+^ T-cell responses; induces high-titer neutralizing antibodies; promotes coordinated humoral and cell-mediated viral clearance.	Complete protection against virulent FPV in kittens Robust humoral and cellular	[6,21,58,59]
DNA Vaccines	Thermostable, easy to produce, rapidly adaptable; enhanced by adjuvants or electroporation.	Induces balanced humoral and cellular immunity; effectively primes cytotoxic CD8^+^ T-cell responses; benefits from molecular adjuvants.	Data limited for FPV; promising results in related parvovirus models; requires optimization for feline use.	[93]
Virus-Like Particle (VLP) Vaccines	Non-infectious, safe, preserves native VP2 conformation; platform versatile for multi-strain/epitope display.	Strong neutralizing antibody responses; activates CD4^+^ and CD8^+^ T cells; promotes durable memory.	Induces durable immunity in kittens; potential for prophylactic and therapeutic use.	[20,23,94]
Probiotic Surface-Displayed Oral Vaccines	Food-grade probiotics (Lactobacillus, etc.); oral admin, high safety; can co-deliver immunostimulatory molecules.	Elicits mucosal sIgA and systemic IgG responses; induces CD4^+^/CD8^+^ T-cell activation; promotes intestinal immune protection.	Animal models show enhanced intestinal protection; VP2 surface display shows potential for FPV; improved viral clearance and long-lasting immunity.	[19]

## Data Availability

The original contributions presented in this study are included in the article. Further inquiries can be directed to the corresponding authors.

## Abbreviations

The following abbreviations are used in this manuscript:

FPV	Feline panleukopenia virus
CPV	Canine parvovirus
CTLs	Cytotoxic T lymphocytes
DC	Dendritic cell
MDA	Maternally derived antibodies
MLV	Modified-live virus
VLP	Virus-like particle
Th1	T helper type 1
Th2	T helper type 2
GALT	Gut-associated lymphoid tissue
IFN	Interferon
Ig	Immunoglobulin
IL	Interleukin
PLA2	Phospholipase A2
TfR1	Transferrin receptor type 1
TfR	Transferrin receptor
nAbs	Neutralizing antibodies
HA	Hemagglutination assay
BEVS	Baculovirus expression vector system
APC	Antigen-presenting cell
MHC	Major histocompatibility complex
PRR	Pattern recognition receptor
WSAVA	World Small Animal Veterinary Association
ABCD	Advisory Board on Cat Diseases
AAHA	American Animal Hospital Association
AAFP	American Association of Feline Practitioners

## References

[1] Awad R.A., Khalil W.K.B., Attallah A.G. (2018). Epidemiology and diagnosis of feline panleukopenia virus in Egypt: Clinical and molecular diagnosis in cats. Vet. World.

[2] Studdert M.J., Kelly C.M., Harrigan K.E. (1973). Isolation of panleucopaenia virus from lions. Vet. Rec..

[3] Huang Q., Zheng Q., Zhou Y., Wang S., Zha L., Chang X. (2026). Therapeutic efficacy of a chimeric neutralizing antibody targeting feline parvovirus. Antivir. Res..

[4] Stuetzer B., Hartmann K. (2014). Feline parvovirus infection and associated diseases. Vet. J..

[5] Kabir A., Habib T., Chouhan C.S., Hassan J., Rahman A., Nazir K. (2023). Epidemiology and molecular characterization of Feline panleukopenia virus from suspected domestic cats in selected Bangladesh regions. PLoS ONE.

[6] Tang A., Li B., Zhu M., Zhu S., Zhang D., Li N., Zhang M., Zhu Y., Li C., Meng C. (2025). A novel feline herpesvirus vector subunit FCV VP1 and FPV VP2 vaccine protects cats against FHV-1 and FPV challenge and induces serum neutralizing antibody responses against FCV. Front. Immunol..

[7] Litster A., Benjanirut C. (2014). Case series of feline panleukopenia virus in an animal shelter. J. Feline Med. Surg..

[8] Addie D.D., Toth S., Thompson H., Greenwood N., Jarrett J.O. (1998). Detection of feline parvovirus in dying pedigree kittens. Vet. Rec..

[9] Truyen U., Addie D., Belak S., Boucraut-Baralon C., Egberink H., Frymus T., Gruffydd-Jones T., Hartmann K., Hosie M.J., Lloret A. (2009). Feline panleukopenia. ABCD guidelines on prevention and management. J. Feline Med. Surg..

[10] Berthier K., Langlais M., Auger P., Pontier D. (2000). Dynamics of a feline virus with two transmission modes within exponentially growing host populations. Proc. Biol. Sci..

[11] Parrish C.R. (1995). Pathogenesis of feline panleukopenia virus and canine parvovirus. Baillieres Clin. Haematol..

[12] Wen Y., Tang Z., Wang K., Geng Z., Yang S., Guo J., Chen Y., Wang J., Fan Z., Chen P. (2024). Epidemiological and Molecular Investigation of Feline Panleukopenia Virus Infection in China. Viruses.

[13] Decaro N., Desario C., Miccolupo A., Campolo M., Parisi A., Martella V., Amorisco F., Lucente M.S., Lavazza A., Buonavoglia C. (2008). Genetic analysis of feline panleukopenia viruses from cats with gastroenteritis. J. Gen. Virol..

[14] Wang Y., Liu Y., Qiao P., Wu H., Liu C., Yang Y., Cao Y., Cui N., Wang L., Huang M. (2025). Evaluating triple inactivated vaccine-induced immunity from a large-scale study in feline population. Sci. Rep..

[15] Horiuchi M., Yamaguchi Y., Gojobori T., Mochizuki M., Nagasawa H., Toyoda Y., Ishiguro N., Shinagawa M. (1998). Differences in the evolutionary pattern of feline panleukopenia virus and canine parvovirus. Virology.

[16] Stone A.E., Brummet G.O., Carozza E.M., Kass P.H., Petersen E.P., Sykes J., Westman M.E. (2020). 2020 AAHA/AAFP Feline Vaccination Guidelines. J. Feline Med. Surg..

[17] Li J., Zeng Y., Peng J., Zhou Y., Li L., Wang Y., Ye Z., Chen Q., Yan Q., Li Q. (2024). Assessing immune evasion potential and vaccine suitability of a feline panleukopenia virus strain. Vet. Vaccine.

[18] Jakel V., Cussler K., Hanschmann K.M., Truyen U., Konig M., Kamphuis E., Duchow K. (2012). Vaccination against Feline Panleukopenia: Implications from a field study in kittens. BMC Vet. Res..

[19] Li J., Zeng Y., Li L., Peng J., Yan Q., Ye Z., Zhang Y., Li W., Cao L., Zhou D. (2024). Development of a recombinant *Lactobacillus plantarum* oral vaccine expressing VP2 protein for preventing feline panleukopenia virus. Vet. Microbiol..

[20] Meng Z., Sun Z., Li J., Qiu W., Wei J., Zhang R., Ji X., Zhu H., Yu J., Liu Y. (2026). Donor Plasmid Optimization Enhances Expression of Feline Parvovirus VP2 Protein in the Baculovirus Expression Vector System. Vaccines.

[21] Yang S., Xia X., Qiao J., Liu Q., Chang S., Xie Z., Ju H., Zou X., Gao Y. (2008). Complete protection of cats against feline panleukopenia virus challenge by a recombinant canine adenovirus type 2 expressing VP2 from FPV. Vaccine.

[22] Feng E., Luo G., Wang C., Liu W., Yan R., Bai X., Cheng Y. (2025). Generation and Immunogenicity of Virus-like Particles Based on the Capsid Protein of a Chinese Epidemic Strain of Feline Panleukopenia Virus. Vet. Sci..

[23] Wang T., Wu H., Wang Y., Guan Y., Cao Y., Wang L., Wang M., Tan F., Pang W., Tian K. (2025). Virus-like Particle Vaccine for Feline Panleukopenia: Immunogenicity and Protective Efficacy in Cats. Vaccines.

[24] Li Z., Zhao G., Zhao Z., Li H., Bai X. (2026). Establishment of a reverse genetics system for feline panleukopenia virus and feasibility study of a live vector vaccine. Virus Genes.

[25] Lappin M.R., Veir J., Hawley J. (2009). Feline panleukopenia virus, feline herpesvirus-1, and feline calicivirus antibody responses in seronegative specific pathogen-free cats after a single administration of two different modified live FVRCP vaccines. J. Feline Med. Surg..

[26] Wu H., Li X., Cui N., Cao Y., Liu C., Ding H., Chen Y., Yang Y., Chen X., Su X. (2025). Novel Strain-Based Triple Inactivated Vaccine Confers Rapid Neutralizing Immunity to Feline Multisystemic Pathogens with Two-Dose Regimen. Transbound. Emerg. Dis..

[27] Digangi B.A., Levy J.K., Griffin B., Reese M.J., Dingman P.A., Tucker S.J., Dubovi E.J. (2012). Effects of maternally-derived antibodies on serologic responses to vaccination in kittens. J. Feline Med. Surg..

[28] Yang M., Jiao Y., Li L., Yan Y., Fu Z., Liu Z., Hu X., Li M., Shi Y., He J. (2024). A potential dual protection vaccine: Recombinant feline herpesvirus-1 expressing feline parvovirus VP2 antigen. Vet. Microbiol..

[29] Chen C., Li Y.L., Lv F.L., Xu L.D., Huang Y.W. (2022). Surface Display of Peptides Corresponding to the Heptad Repeat 2 Domain of the Feline Enteric Coronavirus Spike Protein on Bacillus subtilis Spores Elicits Protective Immune Responses Against Homologous Infection in a Feline Aminopeptidase-N-Transduced Mouse Model. Front. Immunol..

[30] Decaro N., Buonavoglia D., Desario C., Amorisco F., Colaianni M.L., Parisi A., Terio V., Elia G., Lucente M.S., Cavalli A. (2010). Characterisation of canine parvovirus strains isolated from cats with feline panleukopenia. Res. Vet. Sci..

[31] Zochampuii T., Rajkhowa T.K., Jayappa K. (2025). Molecular detection and phylogenetic analysis of feline panleukopenia virus in domestic cat population of Mizoram state, India. Vet. Res. Forum.

[32] Truyen U., Parrish C.R. (2013). Feline panleukopenia virus: Its interesting evolution and current problems in immunoprophylaxis against a serious pathogen. Vet. Microbiol..

[33] Cotmore S.F., Agbandje-McKenna M., Chiorini J.A., Mukha D.V., Pintel D.J., Qiu J., Soderlund-Venermo M., Tattersall P., Tijssen P., Gatherer D. (2014). The family Parvoviridae. Arch. Virol..

[34] Yeo Y.G., Kim H.R., Park J., Kim J.M., Shin Y.K., Lee K.K., Kwon O.K., Jeoung H.Y., Kang H.E., Ku B.K. (2023). Epidemiological and Molecular Approaches for a Fatal Feline Panleukopenia Virus Infection of Captive Siberian Tigers (*Panthera tigris altaica*) in the Republic of Korea. Animals.

[35] Li S., Chen X., Hao Y., Zhang G., Lyu Y., Wang J., Liu W., Qin T. (2022). Characterization of the VP2 and NS1 genes from canine parvovirus type 2 (CPV-2) and feline panleukopenia virus (FPV) in Northern China. Front. Vet. Sci..

[36] Zadori Z., Szelei J., Lacoste M.C., Li Y., Gariepy S., Raymond P., Allaire M., Nabi I.R., Tijssen P. (2001). A viral phospholipase A2 is required for parvovirus infectivity. Dev. Cell.

[37] Pan S., Jiao R., Xu X., Ji J., Guo G., Yao L., Kan Y., Xie Q., Bi Y. (2023). Molecular characterization and genetic diversity of parvoviruses prevalent in cats in Central and Eastern China from 2018 to 2022. Front. Vet. Sci..

[38] Miranda C., Vieira M.J., Silva E., Carvalheira J., Parrish C.R., Thompson G. (2017). Genetic Analysis of Feline Panleukopenia Virus Full-Length VP2 Gene in Domestic Cats Between 2006–2008 and 2012–2014, Portugal. Transbound. Emerg. Dis..

[39] Wu Q., Jin Y., Cao W., Ren Z., Li X., Li Z., Han J., Shi C., Gao R., Yan M. (2025). Engineering a recombinant VP2-Based neutralizing epitope vaccine candidate against canine parvovirus: A preliminary immunogenicity assessment. Vet. Res. Commun..

[40] Kamstrup S., Langeveld J., Botner A., Nielsen J., Schaaper W.M., Boshuizen R.S., Casal J.I., Hojrup P., Vela C., Meloen R. (1998). Mapping the antigenic structure of porcine parvovirus at the level of peptides. Virus Res..

[41] Xie Q., Sun Z., Xue X., Pan Y., Zhen S., Liu Y., Zhan J., Jiang L., Zhang J., Zhu H. (2024). China-origin G1 group isolate FPV072 exhibits higher infectivity and pathogenicity than G2 group isolate FPV027. Front. Vet. Sci..

[42] Liu C., Si F., Li H., Gao J., Sun F., Liu H., Yi J. (2023). Identification and Genome Characterization of Novel Feline Parvovirus Strains Isolated in Shanghai, China. Curr. Issues Mol. Biol..

[43] Mostl K., Egberink H., Addie D., Frymus T., Boucraut-Baralon C., Truyen U., Hartmann K., Lutz H., Gruffydd-Jones T., Radford A.D. (2013). Prevention of infectious diseases in cat shelters: ABCD guidelines. J. Feline Med. Surg..

[44] Decaro N., Buonavoglia C. (2012). Canine parvovirus—A review of epidemiological and diagnostic aspects, with emphasis on type 2c. Vet. Microbiol..

[45] Cotmore S.F., Tattersall P. (2014). Parvoviruses: Small Does Not Mean Simple. Annu. Rev. Virol..

[46] Callaway H.M., Feng K.H., Lee D.W., Allison A.B., Pinard M., McKenna R., Agbandje-McKenna M., Hafenstein S., Parrish C.R. (2017). Parvovirus Capsid Structures Required for Infection: Mutations Controlling Receptor Recognition and Protease Cleavages. J. Virol..

[47] Hueffer K., Palermo L.M., Parrish C.R. (2004). Parvovirus infection of cells by using variants of the feline transferrin receptor altering clathrin-mediated endocytosis, membrane domain localization, and capsid-binding domains. J. Virol..

[48] Parker J.S., Murphy W.J., Wang D., O’Brien S.J., Parrish C.R. (2001). Canine and feline parvoviruses can use human or feline transferrin receptors to bind, enter, and infect cells. J. Virol..

[49] Goodman L.B., Lyi S.M., Johnson N.C., Cifuente J.O., Hafenstein S.L., Parrish C.R. (2010). Binding site on the transferrin receptor for the parvovirus capsid and effects of altered affinity on cell uptake and infection. J. Virol..

[50] Maxwell I.H., Maxwell F. (2004). Parvovirus LuIII transducing vectors packaged by LuIII versus FPV capsid proteins: The VP1 N-terminal region is not a major determinant of human cell permissiveness. J. Gen. Virol..

[51] Strassheim M.L., Gruenberg A., Veijalainen P., Sgro J.Y., Parrish C.R. (1994). Two dominant neutralizing antigenic determinants of canine parvovirus are found on the threefold spike of the virus capsid. Virology.

[52] Truyen U., Gruenberg A., Chang S.F., Obermaier B., Veijalainen P., Parrish C.R. (1995). Evolution of the feline-subgroup parvoviruses and the control of canine host range in vivo. J. Virol..

[53] Levine D.S., Woods J.W. (1990). Immunolocalization of transferrin and transferrin receptor in mouse small intestinal absorptive cells. J. Histochem. Cytochem..

[54] Li L., Liu Z., Liang R., Yang M., Yan Y., Jiao Y., Jiao Z., Hu X., Li M., Shen Z. (2024). Novel mutation N588 residue in the NS1 protein of feline parvovirus greatly augments viral replication. J. Virol..

[55] Cotmore S.F., Gottlieb R.L., Tattersall P. (2007). Replication initiator protein NS1 of the parvovirus minute virus of mice binds to modular divergent sites distributed throughout duplex viral DNA. J. Virol..

[56] Garigliany M., Gilliaux G., Jolly S., Casanova T., Bayrou C., Gommeren K., Fett T., Mauroy A., Levy E., Cassart D. (2016). Feline panleukopenia virus in cerebral neurons of young and adult cats. BMC Vet. Res..

[57] Chastant S., Mila H. (2019). Passive immune transfer in puppies. Anim. Reprod. Sci..

[58] Hu L., Esposito J.J., Scott F.W. (1996). Raccoon poxvirus feline panleukopenia virus VP2 recombinant protects cats against FPV challenge. Virology.

[59] Hu L., Ngichabe C., Trimarchi C.V., Esposito J.J., Scott F.W. (1997). Raccoon poxvirus live recombinant feline panleukopenia virus VP2 and rabies virus glycoprotein bivalent vaccine. Vaccine.

[60] Vihinen-Ranta M., Wang D., Weichert W.S., Parrish C.R. (2002). The VP1 N-terminal sequence of canine parvovirus affects nuclear transport of capsids and efficient cell infection. J. Virol..

[61] Gigler A., Dorsch S., Hemauer A., Williams C., Kim S., Young N.S., Zolla-Pazner S., Wolf H., Gorny M.K., Modrow S. (1999). Generation of neutralizing human monoclonal antibodies against parvovirus B19 proteins. J. Virol..

[62] Parrish C.R., Carmichael L.E., Antczak D.F. (1982). Antigenic relationships between canine parvovirus type 2, feline panleukopenia virus and mink enteritis virus using conventional antisera and monoclonal antibodies. Arch. Virol..

[63] Squires R.A., Crawford C., Marcondes M., Whitley N. (2024). 2024 guidelines for the vaccination of dogs and cats—Compiled by the Vaccination Guidelines Group (VGG) of the World Small Animal Veterinary Association (WSAVA). J. Small Anim. Pract..

[64] Day M.J., Horzinek M.C., Schultz R.D., Vaccination Guidelines Group (2010). WSAVA guidelines for the vaccination of dogs and cats. J. Small Anim. Pract..

[65] Nakarin F., Sprenger K.G. (2025). A paradigm shift in simulating affinity maturation to elicit broadly neutralizing antibodies. Front. Immunol..

[66] Saha A., Ghosh Roy S., Dwivedi R., Tripathi P., Kumar K., Nambiar S.M., Pathak R. (2025). Beyond the Pandemic Era: Recent Advances and Efficacy of SARS-CoV-2 Vaccines Against Emerging Variants of Concern. Vaccines.

[67] Ladekjaer-Mikkelsen A.S., Nielsen J. (2002). A longitudinal study of cell-mediated immunity in pigs infected with porcine parvovirus. Viral Immunol..

[68] Feng H., Hu G.Q., Wang H.L., Liang M., Liang H., Guo H., Zhao P., Yang Y.J., Zheng X.X., Zhang Z.F. (2014). Canine parvovirus VP2 protein expressed in silkworm pupae self-assembles into virus-like particles with high immunogenicity. PLoS ONE.

[69] Swain S.L., McKinstry K.K., Strutt T.M. (2012). Expanding roles for CD4(+) T cells in immunity to viruses. Nat. Rev. Immunol..

[70] Casanova J.L., MacMicking J.D., Nathan C.F. (2024). Interferon-gamma and infectious diseases: Lessons and prospects. Science.

[71] Chen S., Zhu H., Jounaidi Y. (2024). Comprehensive snapshots of natural killer cells functions, signaling, molecular mechanisms and clinical utilization. Signal Transduct. Target. Ther..

[72] Schultz R.D., Mendel H., Scott F.W. (1976). Effect of feline panleukopenia virus infection on development of humoral and cellular immunity. Cornell Vet..

[73] Schultz R.D., Scott F.W. (1973). Absence of an immune response after oral administration of attenuated feline panleukopenia virus. Infect. Immun..

[74] Yu Y., Zhou M., Zhang J., Hua Y., Wang L., Liu Y., Liu D., Xia X. (2009). Preparation of the vaccine with inactivated Feline Panleukopenia Virus isolated from tiger and the preliminary application. Wei Sheng Wu Xue Bao.

[75] Vyas J.M., Van der Veen A.G., Ploegh H.L. (2008). The known unknowns of antigen processing and presentation. Nat. Rev. Immunol..

[76] Lo-Man R., Rueda P., Sedlik C., Deriaud E., Casal I., Leclerc C. (1998). A recombinant virus-like particle system derived from parvovirus as an efficient antigen carrier to elicit a polarized Th1 immune response without adjuvant. Eur. J. Immunol..

[77] Lisicka W., Earley Z.M., Sifakis J.J., Erickson S.A., Mattingly J.R., Wu-Woods N.J., Krishnamurthy S.R., Belkaid Y., Ismagilov R.F., Cyster J.G. (2025). Immunoglobulin A controls intestinal virus colonization to preserve immune homeostasis. Cell Host Microbe.

[78] Xu Y., Cui L., Tian C., Zhang G., Huo G., Tang L., Li Y. (2011). Immunogenicity of recombinant classic swine fever virus CD8(+) T lymphocyte epitope and porcine parvovirus VP2 antigen coexpressed by *Lactobacillus casei* in swine via oral vaccination. Clin. Vaccine Immunol..

[79] Kadaoui K.A., Corthesy B. (2007). Secretory IgA mediates bacterial translocation to dendritic cells in mouse Peyer’s patches with restriction to mucosal compartment. J. Immunol..

[80] Rey J., Garin N., Spertini F., Corthesy B. (2004). Targeting of secretory IgA to Peyer’s patch dendritic and T cells after transport by intestinal M cells. J. Immunol..

[81] Siegrist C.A. (2003). Mechanisms by which maternal antibodies influence infant vaccine responses: Review of hypotheses and definition of main determinants. Vaccine.

[82] Wu H., Qiao P., Chen Y., Liu C., Huo N., Ding H., Wang X., Wang L., Xi X., Liu Y. (2024). Cellular and humoral immune responses in cats vaccinated with feline herpesvirus 1 modified live virus vaccine. Front. Vet. Sci..

[83] Spiri A.M., Novacco M., Meli M.L., Stirn M., Riond B., Fogle J.E., Boretti F.S., Herbert I., Hosie M.J., Hofmann-Lehmann R. (2021). Modified-Live Feline Calicivirus Vaccination Elicits Cellular Immunity against a Current Feline Calicivirus Field Strain in an Experimental Feline Challenge Study. Viruses.

[84] Bergmann M., Schwertler S., Speck S., Truyen U., Hartmann K. (2019). Antibody response to feline panleukopenia virus vaccination in cats with asymptomatic retrovirus infections: A pilot study. J. Feline Med. Surg..

[85] Foley J.E., Orgad U., Hirsh D.C., Poland A., Pedersen N.C. (1999). Outbreak of fatal salmonellosis in cats following use of a high-titer modified-live panleukopenia virus vaccine. J. Am. Vet. Med. Assoc..

[86] Day M.J., Horzinek M.C., Schultz R.D., Squires R.A. (2016). Vaccination Guidelines Group (VGG) of the World Small Animal Veterinary Association (WSAVA). WSAVA Guidelines for the vaccination of dogs and cats. J. Small Anim. Pract..

[87] Tham K.M., Studdert M.J. (1987). Antibody and cell mediated immune responses to an inactivated feline panleukopenia virus vaccine. Zentralbl. Vet. B.

[88] Pollock R.V., Carmichael L.E. (1982). Dog response to inactivated canine parvovirus and feline panleukopenia virus vaccines. Cornell Vet..

[89] Hartmann K., Mostl K., Lloret A., Thiry E., Addie D.D., Belak S., Boucraut-Baralon C., Egberink H., Frymus T., Hofmann-Lehmann R. (2022). Vaccination of Immunocompromised Cats. Viruses.

[90] Scott F.W., Geissinger C.M. (1999). Long-term immunity in cats vaccinated with an inactivated trivalent vaccine. Am. J. Vet. Res..

[91] Egberink H., Frymus T., Hartmann K., Mostl K., Addie D.D., Belak S., Boucraut-Baralon C., Hofmann-Lehmann R., Lloret A., Marsilio F. (2022). Vaccination and Antibody Testing in Cats. Viruses.

[92] Sparkes A. (2010). Feline vaccination protocols: Is a consensus emerging?. Schweiz. Arch. Tierheilkd..

[93] Dahiya S.S., Saini M., Kumar P., Gupta P.K. (2012). Immunogenicity of a DNA-launched replicon-based canine parvovirus DNA vaccine expressing VP2 antigen in dogs. Res. Vet. Sci..

[94] Zhao C., Ao Z., Yao X. (2016). Current Advances in Virus-like Particles as a Vaccination Approach against HIV Infection. Vaccines.

[95] Liu M.A. (2011). DNA vaccines: An historical perspective and view to the future. Immunol. Rev..

[96] Wolff J.A., Malone R.W., Williams P., Chong W., Acsadi G., Jani A., Felgner P.L. (1990). Direct gene transfer into mouse muscle in vivo. Science.

[97] Garcia-Valtanen P., Yeow A.E.L., Mekonnen Z.A., Whelan D.M., Santos R., Al-Delfi Z., Rodrigues S., Gavan P., Howard K., Masavuli M.G. (2025). Thermostable unit solid dose formulations for subcutaneous administration of DNA vaccines. Mol. Ther. Nucleic Acids.

[98] Larraga J., Nogales-Altozano P., Gomez-Marcos L., Ruiz S., Loayza F.J., Rivera-Rodriguez A., Louloudes-Lazaro A., Carlon A.B., Rodriguez-Martin D., Alonso A. (2025). Bivalent SARS-CoV-2 spike immunization with non-replicative antibiotic resistance-free DNA vaccine induces immunity to multiple virus variants. Sci. Rep..

[99] Howarth M., Elliott T. (2004). The processing of antigens delivered as DNA vaccines. Immunol. Rev..

[100] Gurunathan S., Klinman D.M., Seder R.A. (2000). DNA vaccines: Immunology, application, and optimization. Annu. Rev. Immunol..

[101] Shedlock D.J., Weiner D.B. (2000). DNA vaccination: Antigen presentation and the induction of immunity. J. Leukoc. Biol..

[102] O’Donovan L.H., McMonagle E.L., Taylor S., Bain D., Pacitti A.M., Golder M.C., McDonald M., Hanlon L., Onions D.E., Argyle D.J. (2005). A vector expressing feline mature IL-18 fused to IL-1beta antagonist protein signal sequence is an effective adjuvant to a DNA vaccine for feline leukaemia virus. Vaccine.

[103] Flingai S., Czerwonko M., Goodman J., Kudchodkar S.B., Muthumani K., Weiner D.B. (2013). Synthetic DNA vaccines: Improved vaccine potency by electroporation and co-delivered genetic adjuvants. Front. Immunol..

[104] Dunham S.P., Flynn J.N., Rigby M.A., Macdonald J., Bruce J., Cannon C., Golder M.C., Hanlon L., Harbour D.A., Mackay N.A. (2002). Protection against feline immunodeficiency virus using replication defective proviral DNA vaccines with feline interleukin-12 and -18. Vaccine.

[105] Jiao C., Zhang H., Liu W., Jin H., Liu D., Zhao J., Feng N., Zhang C., Shi J. (2020). Construction and Immunogenicity of Virus-Like Particles of Feline Parvovirus from the Tiger. Viruses.

[106] Ahmadivand S., Gomez-Casado E. (2026). Advances in Nanoparticles as Vaccine Adjuvants. Vaccines.

[107] Zachova K., Bartheldyova E., Hubatka F., Krupka M., Odehnalova N., Turanek Knotigova P., Vaskovicova N., Sloupenska K., Hromadka R., Paulovicova E. (2024). The immunogenicity of p24 protein from HIV-1 virus is strongly supported and modulated by coupling with liposomes and mannan. Carbohydr. Polym..

[108] Su K., Ren J., Zhang Y., Yuan C., Wang Y., Yang L., Fu L., Fan T., Song Q. (2025). Intestinal mucosal immune responses induced by oral administration of chitosan nanoparticles encapsulating the PEDV S1 protein. Vet. Res..

[109] Ebrahimian M., Hashemi M., Maleki M., Abnous K., Hashemitabar G., Ramezani M., Haghparast A. (2016). Induction of a balanced Th1/Th2 immune responses by co-delivery of PLGA/ovalbumin nanospheres and CpG ODNs/PEI-SWCNT nanoparticles as TLR9 agonist in BALB/c mice. Int. J. Pharm..

[110] Jiao C., Jin H., Zhang M., Liu D., Huang P., Bai Y., Dai J., Zhang H., Li Y., Wang H. (2023). A bacterium-like particle vaccine displaying protective feline herpesvirus 1 antigens can induce an immune response in mice and cats. Vet. Microbiol..

[111] Puccetti M., Pariano M., Schoubben A., Ricci M., Giovagnoli S. (2024). Engineering carrier nanoparticles with biomimetic moieties for improved intracellular targeted delivery of mRNA therapeutics and vaccines. J. Pharm. Pharmacol..

[112] Xu C., Aqib A.I., Fatima M., Muneer S., Zaheer T., Peng S., Ibrahim E.H., Li K. (2024). Deciphering the Potential of Probiotics in Vaccines. Vaccines.

[113] Chowdhury M.R., Islam A., Yurina V., Shimosato T. (2026). Genetically Modified Lactic Acid Bacteria: A Promising Mucosal Delivery Vector for Vaccines. Probiotics Antimicrob. Proteins.

[114] Wang M., Gao Z., Zhang Y., Pan L. (2016). Lactic acid bacteria as mucosal delivery vehicles: A realistic therapeutic option. Appl. Microbiol. Biotechnol..

[115] Kunisawa J., Kurashima Y., Kiyono H. (2012). Gut-associated lymphoid tissues for the development of oral vaccines. Adv. Drug Deliv. Rev..

[116] Wang X., Wang L., Huang X., Ma S., Yu M., Shi W., Qiao X., Tang L., Xu Y., Li Y. (2017). Oral Delivery of Probiotics Expressing Dendritic Cell-Targeting Peptide Fused with Porcine Epidemic Diarrhea Virus COE Antigen: A Promising Vaccine Strategy against PEDV. Viruses.

[117] Day M.J. (2007). Vaccine safety in the neonatal period. J. Comp. Pathol..

[118] Yu Z., Huang Z., Sao C., Huang Y., Zhang F., Ma G., Chen Z., Zeng Z., Qiwen D., Zeng W. (2013). Oral immunization of mice using Bifidobacterium longum expressing VP1 protein from enterovirus 71. Arch. Virol..

[119] Ma S., Wang L., Huang X., Wang X., Chen S., Shi W., Qiao X., Jiang Y., Tang L., Xu Y. (2018). Oral recombinant Lactobacillus vaccine targeting the intestinal microfold cells and dendritic cells for delivering the core neutralizing epitope of porcine epidemic diarrhea virus. Microb. Cell Fact..

[120] Vilander A.C., Shelton K., LaVoy A., Dean G.A. (2023). Expression of E. coli FimH Enhances Trafficking of an Orally Delivered *Lactobacillus acidophilus* Vaccine to Immune Inductive Sites via Antigen-Presenting Cells. Vaccines.

[121] Leontieva G., Kramskaya T., Gupalova T., Bormotova E., Desheva Y., Korzhevsky D., Kirik O., Koroleva I., Borisevitch S., Suvorov A. (2024). Comparative Efficacy of Parenteral and Mucosal Recombinant Probiotic Vaccines Against SARS-CoV-2 and S. pneumoniae Infections in Animal Models. Vaccines.

[122] Lin J., Mou C., Zhang S., Zhu L., Li Y., Yang Q. (2022). Immune Responses Induced by Recombinant Bacillus subtilis Expressing the PEDV Spike Protein Targeted at Microfold Cells. Vet. Sci..

[123] Liu Y., Cao X., Liu H., Zhang W. (2025). The crosstalk between probiotics and T cell immunity. Front. Immunol..

[124] Redenti A., Im J., Redenti B., Li F., Rouanne M., Sheng Z., Sun W., Gurbatri C.R., Huang S., Komaranchath M. (2024). Probiotic neoantigen delivery vectors for precision cancer immunotherapy. Nature.

[125] Ashaolu T.J., Greff B., Varga L. (2025). Action and immunomodulatory mechanisms, formulations, and safety concerns of probiotics. Biosci. Microbiota Food Health.

[126] Abavisani M., Ebadpour N., Khoshrou A., Sahebkar A. (2024). Boosting vaccine effectiveness: The groundbreaking role of probiotics. J. Agric. Food Res..

[127] Moradi-Kalbolandi S., Majidzadeh-A K., Abdolvahab M.H., Jalili N., Farahmand L. (2021). The Role of Mucosal Immunity and Recombinant Probiotics in SARS-CoV2 Vaccine Development. Probiotics Antimicrob. Proteins.

[128] Xu R., Hong H.A., Khandaker S., Baltazar M., Allehyani N., Beentjes D., Prince T., Ho Y.L., Nguyen L.H., Hynes D. (2025). Nasal delivery of killed Bacillus subtilis spores protects against influenza, RSV and SARS-CoV-2. Front. Immunol..

[129] Atalay E., Sozdutmaz I., Kokkaya S. (2026). Development of a recombinant Lactobacillus casei strain expressing Bovine rotavirus VP4 and VP7 proteins as an oral vaccine candidate against calf enteritis. Vet. Ital..

[130] Chen M., Cao Z., Chen Y., Ren Z., Lu S., Pu D., Zhang S., Zhang G., Yang J., Pu J. (2025). Immunomodulatory effects of probiotic Lactobacillus brevis ZG2488 on SARS-CoV-2 vaccine responses in mice. Front. Microbiol..

[131] Liu F., Chang J., Huang J., Liao Y., Deng X., Guo T., Kong J., Kong W. (2025). Surface Display of Avian H5 and H9 Hemagglutinin Antigens on Non-Genetically Modified Lactobacillus Cells for Bivalent Oral AIV Vaccine Development. Microorganisms.

[132] Guo T., Gao C., Hao J., Lu X., Xie K., Wang X., Li J., Zhou H., Cui W., Shan Z. (2022). Strategy of Developing Oral Vaccine Candidates Against Co-Infection of Porcine Diarrhea Viruses Based on a Lactobacillus Delivery System. Front. Microbiol..

[133] Niewiesk S. (2014). Maternal antibodies: Clinical significance, mechanism of interference with immune responses, and possible vaccination strategies. Front. Immunol..

[134] Jenkins E., Davis C., Carrai M., Ward M.P., O’Keeffe S., van Boeijen M., Beveridge L., Desario C., Buonavoglia C., Beatty J.A. (2020). Feline Parvovirus Seroprevalence Is High in Domestic Cats from Disease Outbreak and Non-Outbreak Regions in Australia. Viruses.

[135] Su X., Zhou H., Jiang W., Xu F., Xiao B., Zhang J., Qi Q., Yang B. (2026). Isolation and evolutionary analysis of feline panleukopenia virus strains FPV-BJ-J2 and FPV-BJ-J3 (T440A, N564S, A568G) in Beijing, China. Front. Vet. Sci..

[136] Safwat M.S., Xi C., El-Sayed M S., Anwer A.Z., Ali M.E., Abdallah E.S.A., Amer H.M., Saeed O.S., Khalil G.M., Abdelhaleem S.W. (2025). Emergence, surge, and fading of the novel feline parvovirus Thr390Ala mutant in Egyptian cats during 2023: Insights from a comprehensive full-length VP2 genetic analysis. BMC Vet. Res..

